# Rapid updating of ecological information in wild Guinea baboons

**DOI:** 10.1007/s10071-026-02119-4

**Published:** 2026-10-30

**Authors:** Lisa Ohrndorf, Roger Mundry, Dietmar Zinner, Julia Fischer

**Affiliations:** 1 Georg-August-Universität Göttingen, Johann-Friedrich-Blumenbach Institute, Department for Primate Cognition, Kellnerweg 4, 37077 Göttingen, Germany; 2 German Primate Center - Leibniz Institute for Primate Research, Cognitive Ethology Laboratory, Kellnerweg 4, 37077 Göttingen, Germany; 3 Leibniz ScienceCampus Primate Cognition, Kellnerweg 4, 37077 Göttingen, Germany

**Keywords:** Goal-directed foraging, Feeding ecology, *Papio papio*, Spatial cognition, Travel routes

## Abstract

Knowing where to find what kind of food, and when, is essential for survival and reproduction. Studying animals’ foraging strategies thus provides insights into the cognitive processes underlying real-world problem solving. We investigated whether free-ranging Guinea baboons (*Papio papio*) keep track of the spatio-temporal availability of a preferred food resource, the Natal orange (*Strychnos spinosa*). An exceptional wildfire that destroyed most ripe fruits in 2019 provided a quasi-experimental opportunity to assess whether Guinea baboons flexibly adjusted their space use to an unexpected change in resource availability or instead continued to follow habitual routines. The animals’ space use patterns shifted significantly following the wildfire. After one single visit, they stopped going to the area during the period when Natal oranges would normally be available. In the following year, when Natal oranges were available again, the baboons resumed regular space use patterns. We also assessed travel linearity, speed, and departure time from sleeping sites towards the target area with and without ripe fruit. Guinea baboons travelled more linearly, 14% faster, and ca. 40 min earlier toward the target area when *S. spinosa* was in season than when it was not. In conclusion, Guinea baboons rapidly adjusted their space use patterns in response to changes in the availability of a high-value food resource. These results underscore their capacity for flexible, reward-sensitive decision-making and goal-directed foraging.

**Supplementary Information:**

The online version contains supplementary material available at https://doi.org/10.1007/s10071-026-02119-4.

## Introduction

Knowing ‘where’ to find ‘what’, ‘when’ it is available, and how to move between specific locations is essential to maximise foraging outcomes while simultaneously minimising associated costs. Navigational capacities can thus have important implications for the survival and reproduction of animals. Recent research has revived interest in foraging cognition as a central driver of cognitive evolution (DeCasien et al. 2017; Rosati 2017; Bouret et al. 2024; Keum et al. 2025). One way animals may forage efficiently is by memorising the locations of valuable resources and travelling directly towards these out-of-sight goals (Trapanese et al. 2019; Salmi et al. 2020). Such ‘goal-directedness’ is often inferred from observed movement patterns such as increased travel linearity, faster approaches, or earlier departures towards high-value feeding sites (Noser & Byrne 2007a; Janmaat et al. 2014; Salmi et al. 2020).

Several studies across taxa have highlighted animals’ abilities to optimise travel paths towards high-value resources, frequently demonstrating spatial memory. Insects such as fruit flies (*Drosophila melanogaster*), honeybees (*Apis mellifera*), and bumblebees (*Bombus* spp.) demonstrated robust spatial learning abilities and efficient traplining between food resources (Menzel et al. 2005; Osborne et al. 2008; Ofstad et al. 2011; Reynolds et al. 2013). Birds have long served as key models for studying the navigational capacity of animals (Wallraff 2005), from homing pigeons (*Columba livia*) to a wide range of other species such as honey buzzards (*Pernis ptilorhynchus)* (Sugasawa & Higuchi 2019), cuckoos (*Cuculus canoris*) (Thorup et al. 2020) and rufous hummingbirds (*Rufus selasphorus*) (Healy et al. 2022), with research highlighting the complementary use of cue-based and memory-based navigation (Menzel & Fischer 2011; Kashetsky et al. 2021). Among primates, traplining behaviour has been demonstrated experimentally in several strepsirrhine (e.g., fat-tailed dwarf lemurs, *Cheirogaleus medius*; grey mouse lemurs, *Microcebus murinus*; aye-ayes, *Daubentonia madagascariensis*) and cercopithecine species (e.g., vervet monkeys, *Chlorocebus pygerythrus*; Japanese macaques, *Macaca fuscata*) (Kumpan et al. 2022). Further, western gorillas (*Gorilla gorilla gorilla*), chacma baboons (*Papio ursinus*), and western chimpanzees (*Pan troglodytes verus*) show space use patterns consistent with goal-directed foraging, including linear travel, selective bypassing of low-quality food resources, and earlier departure towards valuable feeding trees (Noser & Byrne 2007b; Janmaat et al. 2014; Salmi et al. 2020).

Yet, observed space use patterns alone are often insufficient to differentiate between habitual and goal-directed behaviour. Linearity, for example, may arise not only from deliberate targeting of a known resource but also from landscape constraints or simple rules such as avoiding backtracking (Janmaat et al. 2021). Similarly, faster travel or earlier departures could reflect fixed routines rather than flexible, goal-directed decision-making (Janmaat et al. 2021). This ambiguity is further compounded by differences in how ‘goal-directedness’ is defined across research domains. In spatial cognition, the term ‘goal-directedness’ typically refers to efficient travel toward out-of-sight resources. In the framework of associative dual processing, however, goal-directed decision making denotes actions selected based on their expected outcomes, allowing behaviour to be flexibly adjusted when outcome values or action-outcome contingencies change. In contrast, habitual behaviour is governed by learned stimulus–response associations that may persist irrespective of current payoffs (de Wit & Dickinson 2009; Balleine & Dezfouli 2019). In a foraging context, both approaches likely capture complementary aspects of the same functional principle: animals adjust their space use to maximise rewards and minimise costs. Goal-directedness may thus be expressed both as efficient, directed travel towards a localised food resource, and as outcome-sensitive adjustments of space use when payoffs shift. Here, we use this overlap to interpret observed movement patterns as indicators of flexible, reward-sensitive decision-making and goal-directed foraging.

**Fig. 1:**
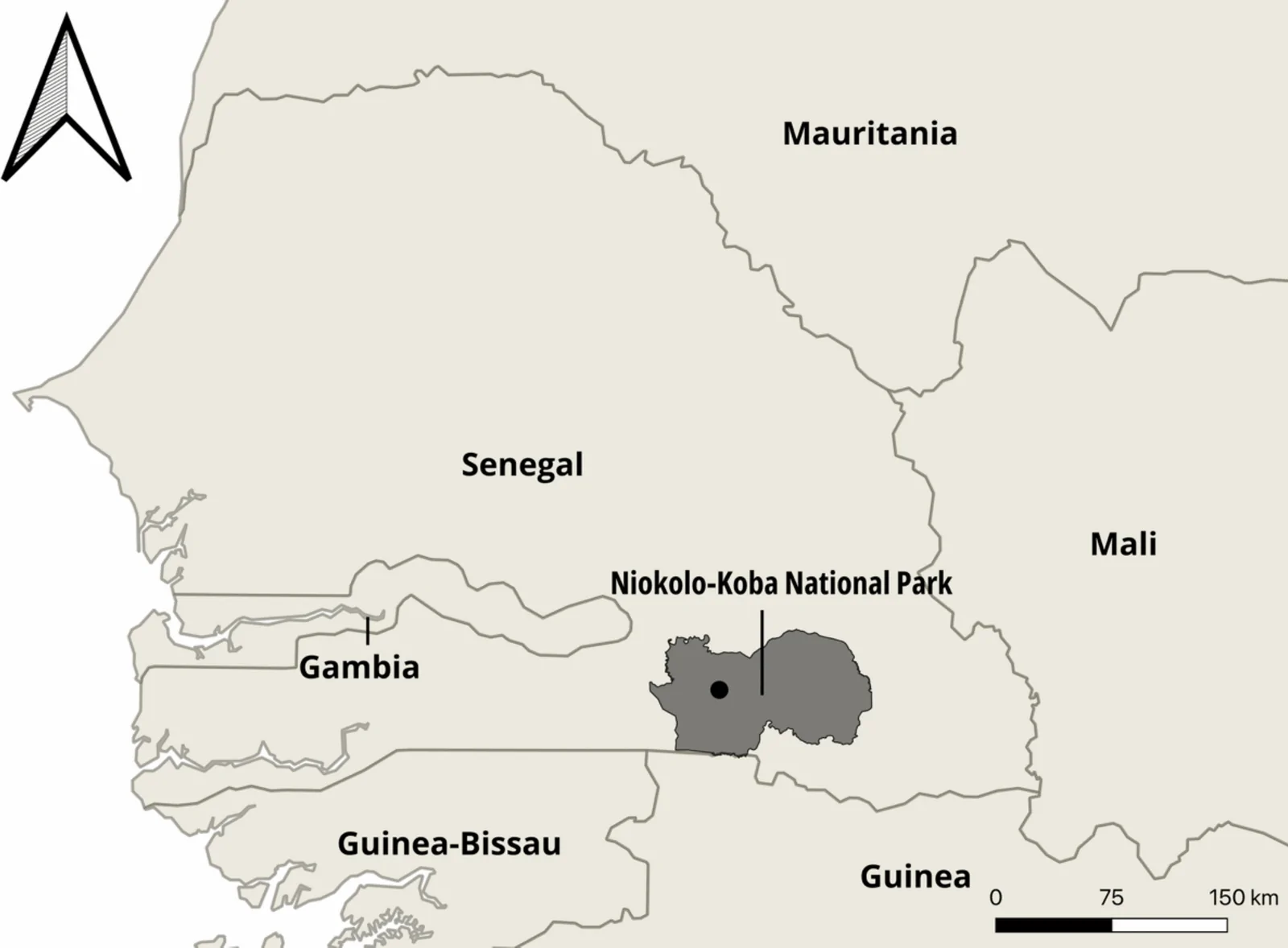
Location of the field site of the CRP Simenti (black dot) within the Niokolo-Koba National Park (PNNK)

In the present study, we analysed Guinea baboon (*Papio papio*) movement patterns towards a valuable, localised, and temporally available food resource, the Natal orange (*Strychnos spinosa*). The trees typically bear ripe fruit in the dry season from January to March. During this time, it is among the most frequently consumed food items. Based on behavioural observations from 2022 and 2023, they accounted for 18.2% of total feeding time in February (Table S1). The orange-sized fruits are enclosed in a firm shell and contain tightly packed seeds surrounded by a layer of fleshy, edible pulp. Of particular interest was how the baboons’ space use patterns changed when this food resource was unexpectedly not available during the typical season. At our long-term field site, Centre de Recherche du Primatologie Simenti in the Niokolo Koba National Park in south-east Senegal (Fischer et al. 2017) (Fig. 1), the Natal orange is one of the few feeding tree species that provide fleshy fruit outside the rainy season (Zinner et al. 2021; Table S1)*.*

To shed light on the ‘cognitive processing architecture’ (Dickinson 2012) underlying the animals’ space use, that is, the distinction between habitual and goal-directed decision-making, we made use of a natural experiment that occurred in the dry season of 2019. In late January 2019, the National Park experienced bushfires that had an unusually strong effect on the availability of fruits of *S. spinosa*. While such fires are a regular feature of the landscape and the Park’s management regime (Nielsen et al. 2003), they typically affect only herbaceous vegetation, clearing tall grass from the preceding rainy season. In that year, however, the fires were exceptional in timing and progression, and nearly all ripe fruits of *S. spinosa* were destroyed by the heat. The trees themselves were only lightly affected, and larger trees of other species remained unharmed.

This unique event temporarily removed an important food resource, allowing us to assess whether the baboons shifted their space use patterns in 2019 compared to two years prior and two years after the destruction of fruits by bushfires. If the baboons’ movement patterns are predominantly habitual, we would expect them to continue following their usual routines in 2019, despite the absence of ripe fruit. In contrast, if their movement patterns are goal-directed and reward-sensitive, they should learn from inspecting the area that the fruit was not available (and would also not become available later), adjust their space use after a few unsuccessful visits and stop returning to the area.

We further assessed the linearity and speed of travel, as well as departure time from sleeping sites toward the target area, when ripe fruit was available compared with when it was not. If the baboons keep track of the availability of this food resource, we expect them to depart from sleeping trees earlier and travel more linearly and faster when Natal oranges are available.

## Material and methods

### Study site

Data collection took place at the Centre de Recherche de Primatologie (CRP), Simenti (13°01 ‘34 ‘‘ N, 13°17 ‘41 ‘‘ W) in the Niokolo Koba National Park (PNNK) in South-East Senegal (Fig. 1). The study site is part of the Sudanian and Sahelo-Sudanian climatic zone with pronounced seasonality and high variability of rainfall among seasons (Arbonnier 2002; Alberts et al. 2005). The rainy season typically lasts from June to October, with May and mid-October constituting transitional periods with little and very variable rainfall (Arbonnier 2002). The average annual precipitation from 2015 to 2023 in Simenti was 932 mm. Temperatures ranged from 10 °C to 53 °C in the dry season and from 21 °C to 49 °C in the rainy season. The vegetation represents a mosaic of grasslands, wooded savannahs, and gallery forests along streams or larger water bodies (Arbonnier 2002; Burgess et al. 2004). During the rainy season, vegetation density increases substantially, with tall grasses growing predominantly in wetlands and savannah habitats. Seasonal flooding affects wetlands and some smaller streams draining from them. While flooded wetlands can restrict movement, the smaller streams generally do not represent permanent barriers at the study site. The overall landscape is relatively open and lacks major topographical boundaries such as cliffs or steep hills that could constrain terrestrial movement. The Gambia River constitutes the main boundary of the study site to the south. Several habituated parties of Guinea baboons live at the study site and have been followed extensively by researchers since 2007 (Fischer et al. 2017). For more information on the study site, see Fischer et al. (2017) and Zinner et al. (2021).

## Study species

Guinea baboons occupy a variety of habitats, from moist forests and mangroves in Guinea to semi-deserts in Mauritania, and are likely exposed to considerable seasonal variation in precipitation across their range (Winder 2014; Chala et al. 2019; Zinner et al. 2021). They are predominantly terrestrial and live in large groups comprising 20 to more than 300 individuals with a nested multi-level social organisation and female-biased dispersal (Patzelt et al. 2011; Kopp et al. 2015; Fischer et al. 2017). The first level of social organisation is the unit consisting of one adult male, one to several females, their dependent offspring and occasionally secondary males (Goffe et al. 2016). Several of these units form parties which then, together with other parties, form gangs (Fischer et al. 2017). Events of fissions and fusions in Guinea baboons typically happen along consistent, predictable lines (i.e., molecular fission–fusion dynamics, see Grueter et al. 2012, 2020), and most frequently at the level of parties. Parties frequently associate with other parties and often stay in close spatial proximity to neighbouring parties throughout the day (Ohrndorf et al. 2025a).

For this study, we investigated space use patterns of seven habituated Guinea baboon parties (P5, P6, P6I, P6W, P9, P9B, P13), which represent a subset of the local population. Party membership is assigned based on spatial proximity, and individuals spend most of their time with members of their parties (Patzelt et al. 2014; Ohrndorf et al. 2025a, b). Our study parties averaged 29 individuals (range 6 to 51) during the study period, including adults and immatures. On average, parties comprised 5 adult males, 7 adult females, 3 subadults, and 14 juveniles. The home ranges of parties in the Niokolo-Koba National Park span 20 to 50 km^2^ on average (Sharman 1982; Zinner et al. 2021) with considerable overlap between them (Ohrndorf et al. 2025a). Guinea baboon parties at the study site predominantly sleep in the gallery forest surrounding the Gambia River (Ohrndorf et al. 2025b).

Like other baboon species, Guinea baboons are considered eclectic omnivores with a high proportion of fruit in their diet (Sharman 1982; Zinner et al. 2021). At the study site, the Natal orange (*S. spinosa*) is one of the few feeding tree species that provide fleshy fruit outside the rainy season (Zinner et al. 2021; Table S1)*.* Trees of this species occur predominantly in the northern parts of our study parties' home ranges (Figures S1, S2) and are therefore spatially separated and generally out of sight from most sleeping sites. *S. spinosa* typically bears ripe fruit from January to March, which the baboons feed on preferentially during this time (Table S1).

## Data collection

### Target area

**Fig. 2:**
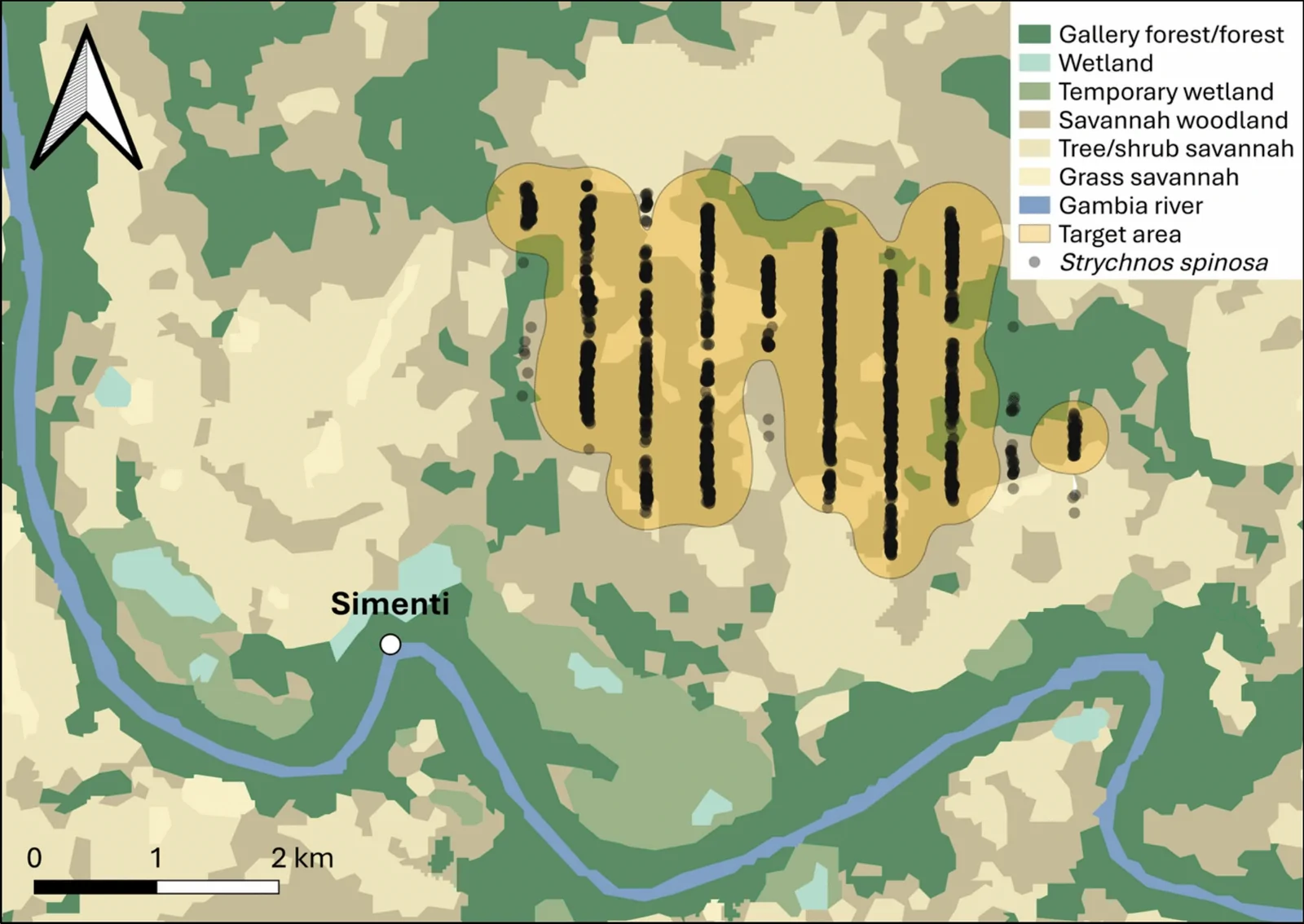
Location of the ‘Target area’ representing the occurrence of *S. spinosa* trees at high densities as mapped in 2023 along 10 north–south transects (within 5 m on either side; 5847 trees recorded in total, 277 trees per hectare, delineated using kernel density estimation (KDE) at the 95% contour level)

To delineate the area in which *S. spinosa* occurs at a high density, we established 10 parallel transects in a North–South direction of roughly 4 km length with 500 m between transects in 2023. We noted the number and locations of all *S. spinosa* trees within 5 m of the transect line. We ran a kernel density estimation in QGIS 3.34.10 (QGIS.org 2023), using the heatmap function with a quartic kernel shape and a radius/bandwidth of 500 m. We used the 95% contour level to delineate the ‘target area’ (Fig. 2). Smaller bandwidths or lower contour levels produced artificial ‘islands’ due to the spacing between our transects, which would have resulted in an underestimation of the spatial extent of the target area. Based on our observations, the area is relatively continuously vegetated with *S. spinosa*, and the selected bandwidth and contour level therefore better reflect the continuous high-density distribution of this food resource. The target area was almost completely enclosed in all of the study parties’ home ranges (Figure S2).

### Season of *S. spinosa*

We established when ripe fruits of *S. spinosa* were most abundant using phenological data from 2013 to 2014 and from 2022 to 2023 (Klapproth, unpublished data; Ohrndorf et al. 2025b). Although fruits are already available in December, they only start to ripen in January, and ripe fruits are most abundant in February, with some lasting until March. We therefore defined the “season” as January to March of each year. We classified all remaining months as “non-season”. We also classified February and March 2019 as non-season because most ripe fruit had burned.

### Fire regime and impact

To illustrate the fire regime of the study area and assess potential interannual variation, we quantified the burned area within the target area using the MODIS burned-area product (MCD64A1) from October 2014 to May 2023 (Giglio et al. 2021). Given the product’s spatial resolution of 463.31 m, we added a 500 m buffer around the target area to ensure we captured all potentially burned MODIS cells intersecting the target area. For each year, we defined the dry season from October 15 of the previous year to May 31. Within each dry season, we then extracted the number of burned MODIS cells and the timing of burning events relative to the onset of the dry season (i.e., days since October 15), including associated uncertainty estimates.

To further investigate potential impacts of the annual fires on the plant productivity of the following rainy season, we extracted average 10-day NDVI values for the target area and a surrounding 500 m buffer (13.0263 to 13.0662° N, 13.2907 to 13.2346° W) from 2014 to 2023 from version 3 of the Copernicus Global Land Service (Copernicus Global Land Service 2020).

### GPS data

Locational data of Guinea baboon parties were collected through direct observation during half-day follows (typically from 07:00 to 13:00 h) using Garmin handheld GPS devices (GARMIN GPSmap 62-66 s) from April 2014 to March 2023. Only one researcher collected locational data per party and day. The initial waypoint for a given day was taken upon first contact with a specific party, and, the last waypoint was taken at the end of baboon contact. During the follows, party-waypoints were taken at 30-min intervals by one researcher, each waypoint representing the location of this researcher following one adult or subadult individual of the study party. The researcher periodically switched between individuals to ensure even coverage of the party. In the event of a within-party fission (n=3), the researcher continued to follow the individual they were observing and subsequently switched only among individuals within that subgroup. The resulting data reflect the party’s overall movement during half-day follows. We only included tracks that were clearly assignable to one study party in the analyses.

Although half-day follows ended at around 13:00 h, frequent afternoon visits to the target area are unlikely. GPS collar data indicate that the vast majority of first visits to the target area occurred before 14:00 h (Figure S3). Furthermore, because the target area lies in the northern parts of the home ranges and is far from the most frequently used sleeping sites, baboons typically travelled there in the morning, remained there until midday, and returned to sleeping sites in the afternoon (Ohrndorf, personal observation).

### Linearity and speed of travel

To assess whether Guinea baboons travelled more linearly and faster towards the target area when *S. spinosa* fruits were available compared to when they were not, we used a subset of the overall GPS dataset collected between April 2014 and March 2023. We compared baboon space use during the fruiting season of *S. spinosa* (January to March) to space use outside the fruiting season (April to December) of each year. We classified February and March 2019 as non-season due to the loss of ripe fruit following a bushfire.

**Fig. 3:**
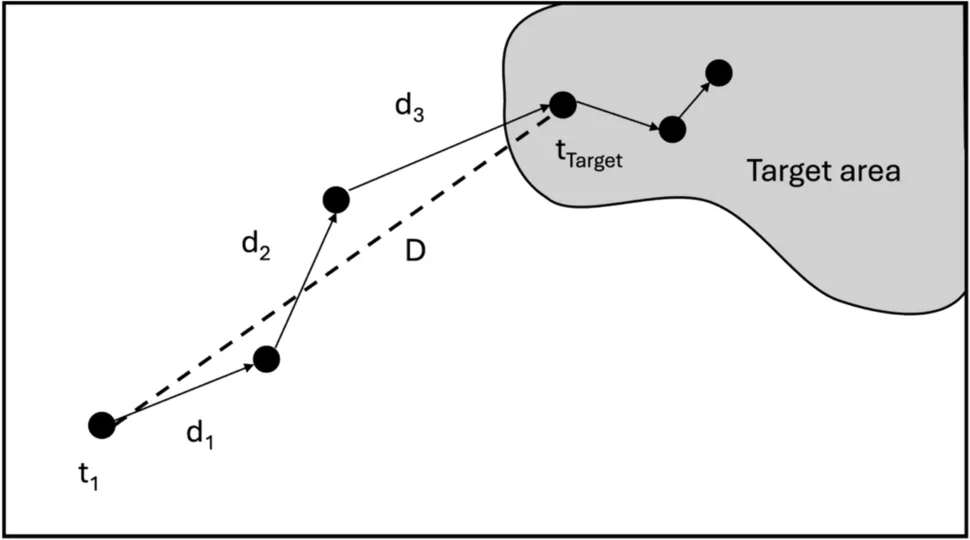
Schematic depiction of calculations for linearity and speed of travel. D = Euclidean distance travelled, d_i_ = distance between successive waypoints, t_1_ = time of the day’s first waypoint (at first contact), t_Target_= time of first waypoint in target area

We considered only days on which the baboons moved toward the target area, i.e., at least one point, corresponding to the location of a researcher following one adult or subadult individual of a given party, fell into the target area. We omitted tracks with two waypoints or fewer before the baboons entered the target area. We did not impose an upper limit on the number of waypoints before entering the target area, as differences in approach duration (i.e., the number of waypoints before entering the target area) capture variation in how parties approached the target area in relation to fruit availability. We then calculated a simple linearity index (1) by dividing the Euclidean distance from the first point of a given day to the first point of that day that was within the target area by the sum of the distances between successive waypoints leading up to the target area (Fig. 3). We calculated the mean travel speed (2) by dividing the sum of the distances between successive waypoints leading up to the target area by the sum of the time lags between the respective waypoints (Fig. 3).

(1) Linearity = $$\frac{D}{d1+d2+\dots +di}$$

(2) Speed= $$\frac{d1+d2+\dots +di}{{t}_{Target}-{t}_{1}}$$

We took waypoints only every 30 min following our general routine, whereas most other comparable studies chose shorter sampling intervals of 1 min (Normand & Boesch 2009), 2 min (Valero & Byrne 2007), 5 min (Noser & Byrne 2007b; Salmi et al. 2020; Fei et al. 2023). However, 10-min (Tujague & Janson 2017; Hanzawa et al. 2024; Cheng et al. 2025) or 15-min intervals (Schreier & Grove 2014; Lewis-Bevan et al. 2025) have also been used. Because of the coarse sampling, we likely underestimated the actual distance walked. Sennhenn-Reulen et al. (2017) found an underestimation of ca. 25% of the actual travel distance at a 30-min sampling interval in the same study population. As a result, our linearity values are likely overestimated relative to the true linearity of baboon travel, and travel speed is likely underestimated. Nonetheless, as our analyses are based on internal comparisons and modelling within this dataset, the observed patterns and relative effects are robust to the sampling interval.

### Time of departure from sleeping sites

We defined the time of departure from sleeping sites as the time at which the researcher’s GPS track, recorded while following the party, crossed a 300 m radius around the sleeping site. In this analysis, we included only days when we found the baboons at their sleeping sites. We then calculated the relative time of departure from the sleeping site as the time difference between sunrise and the time the baboons left the sleeping site. We derived times of sunrise for the study area using the package *suncalc* in R (Thieurmel et al. 2019).

### Temperature

Because temperature may affect baboon departure from the sleeping site (e.g., later departure due to sunbathing on cold mornings), we controlled for its effect on departure time. We recorded daily minimum and maximum temperatures using a digital thermometer (Eurochrom ETH 5500 Thermometer/Hygrometer) positioned outdoors at the campsite but protected from direct sunlight and precipitation. Like most of the Guinea baboons’ sleeping sites, the campsite is located in a cleared patch in the gallery forest bordering the Gambia River. Thus, we assume that campsite temperatures provide a reasonable proxy for the ambient conditions experienced by the study parties at their sleeping sites.

## Statistical analyses

### Natural experiment

We assessed changes in space use patterns in response to pronounced bushfires in the study area in 2019 using data from January to March from 2015 to 2023. No data were available for the 2014 fruiting season, as systematic long-term data collection only began in April 2014, after the fruiting season of *S. spinosa*. We calculated the number of days per month on which at least one waypoint for a study party fell within the target area and compared the relative frequency of visits to the target area during the season of *S. spinosa* across years.

Because these unusual bushfires occurred only once during the study period, this ‘natural experiment’ effectively yielded one data point against which other years could be compared. We therefore were unable to perform elaborate statistical modelling and instead focus on descriptive comparisons of visit frequencies. Nonetheless, this rare event provided a unique opportunity to examine how free-ranging Guinea baboons update expectations and adapt their foraging strategies in response to real-world environmental unpredictability.

### Linearity of travel (Model 1)

To estimate how travel linearity toward the target area differed between season and non-season across the study period (2014 to 2023), we fitted a generalized linear mixed model (GLMM; Baayen 2008) with a beta error structure and logit link function (McCullagh & Nelder 1989) using the ‘glmmTMB’ package version 1.1.7 (Brooks et al. 2017) in R version 4.3.1 (R Core Team 2023). We used the linearity index as a response and season (yes/no) as a fixed effect. As we had repeated observations of several different study parties, we included party ID as a random intercept effect. We also included the random slope of season (yes/no) in the model to keep type I error rates at a nominal level of 0.05 (Barr et al. 2013; Schielzeth & Forstmeier 2009). Initially, we also included estimates of the correlations between random intercept and slope in the model. However, model fit did not decrease when removing the correlation from the model (log-likelihoods: full model including correlation: 646.76, df=6; full model without correlation: 646.42, df=5). We therefore continued the analysis with the model lacking the correlation parameter (Table 1).

**Table 1:** Overview of the fitted models comparing linearity, travel speed, and departure time from sleeping sites toward the target area between season (January to March) and non-season (April to December)

Model	Response	Fixed effects	Random effects	Error structure	Sample sizes
1	Linearity	season (yes/no)^a)^	(1 + season (yes/no)||partyID)	Beta	530 (7 parties)
2	Log travel speed	season (yes/no)^a)^	(1 + season (yes/no)||partyID)	Gaussian	530 (7 parties)
3	Relative time of departure from sleeping site^b)^	season (yes/no)^a)^ + z.minimum temperature	(1 + season (yes/no) + z.minimum temperature|partyID)	Gaussian	285 (7 parties)

The analysed data span April 2014 to March 2023. February and March 2019 were classified as non-season due to the loss of ripe fruits following a bushfire

a) Season (yes/no): indicates whether a track was recorded during the fruiting season of *S. spinosa* (January – March).

b) Relative time of departure from sleeping sites: minutes elapsed from sunrise until the party left a 300 m radius around the sleeping site.

### Travel speed and relative time of departure from sleeping sites (Models 2 and 3)

For travel speed and the relative time of departure from the sleeping site, we fitted two linear mixed models (LMM; Baayen 2008) with a Gaussian error structure and identity link function (McCullagh & Nelder 1989) using the function ‘lmer’ of the ‘lme4’ package version 1.1–33.1 (Bates et al. 2015). We log-transformed travel speed to increase the probability of model convergence. We included log speed as the response variable and season (yes/no) as a fixed effect in model 2. For model 3, we included relative time of departure from sleeping sites as the response variable and season (yes/no) as a fixed effect. To control for the effects of temperature on time of departure from sleeping sites, we z-transformed the minimum temperature recorded on a given day to a mean of 0 and a standard deviation of 1 and added it as a fixed effect in model 3. As for model 1, we included party ID as a random intercept effect in both models. We further included the random slope of season (yes/no) in the models to keep type I error rates at a nominal level of 0.05 (Barr et al. 2013; Schielzeth & Forstmeier 2009). Model 3 additionally included the random slope of minimum temperature. As for model 1, we initially included estimates of the correlation between random intercepts and slopes in model 2 and model 3. For model 2, model fit did not decrease when removing the correlation (log-likelihoods: full model including correlation: − 290.23, df=6; full model without correlation: − 290.90, df=5), so we proceeded with the analysis with the model lacking correlations. Model 3 included correlations between random intercept and slopes (Table 1). We used the function ‘lmer’ from the package ‘lmertest’ (version 3.1–3; Kuznetsova et al. 2017) to test the effect of our fixed effects employing the Satterthwaite approximation (Luke 2017).

### General considerations

For model 1, we checked whether the assumption of the absence of overdispersion was met. With a dispersion parameter of 1.16, the response was not overdispersed given the model. After fitting models 2 and 3, we checked whether the assumption of homogeneity and normal distribution of residuals was fulfilled by visually inspecting a QQ-plot (Field 2009) of residuals and residuals plotted against fitted values (Quinn & Keough 2002). These showed that assumptions were met. For all models, we assessed model stability by dropping each party from the data one at a time and comparing the estimates from models fitted to these subsets of data to those derived from the respective model for the full dataset. All models were of good stability (see results). We obtained confidence intervals of model estimates and fitted values using parametric bootstraps (N=1000; functions ‘simulate’ and ‘bootMer’ of the packages ‘glmmTMB’ and ‘lme4’, respectively).

## Results

### Fire regime and impact

The total extent of burned area within the target area was broadly comparable among years, although there was some interannual variation (Figure S4). On average, 60 MODIS cells burned per dry season (range 45 to 92). In 2019, 64 cells burned, indicating that the overall extent of burned area was not exceptional. In contrast, the temporal dynamics of burning in 2019 differed markedly. Whereas burning typically occurred gradually or much earlier in the dry season (mid-December to early January), in 2019 most of the target area burned within a few days in late January (51 MODIS cells between January 27 and 30), coinciding with the onset of the *S. spinosa* fruiting season (Figure S4). NDVI values during the subsequent rainy season were comparable to those observed before and after the 2019 bushfires, indicating no pronounced differences in overall vegetation productivity (Figure S5).

### Natural experiment

**Fig. 4:**
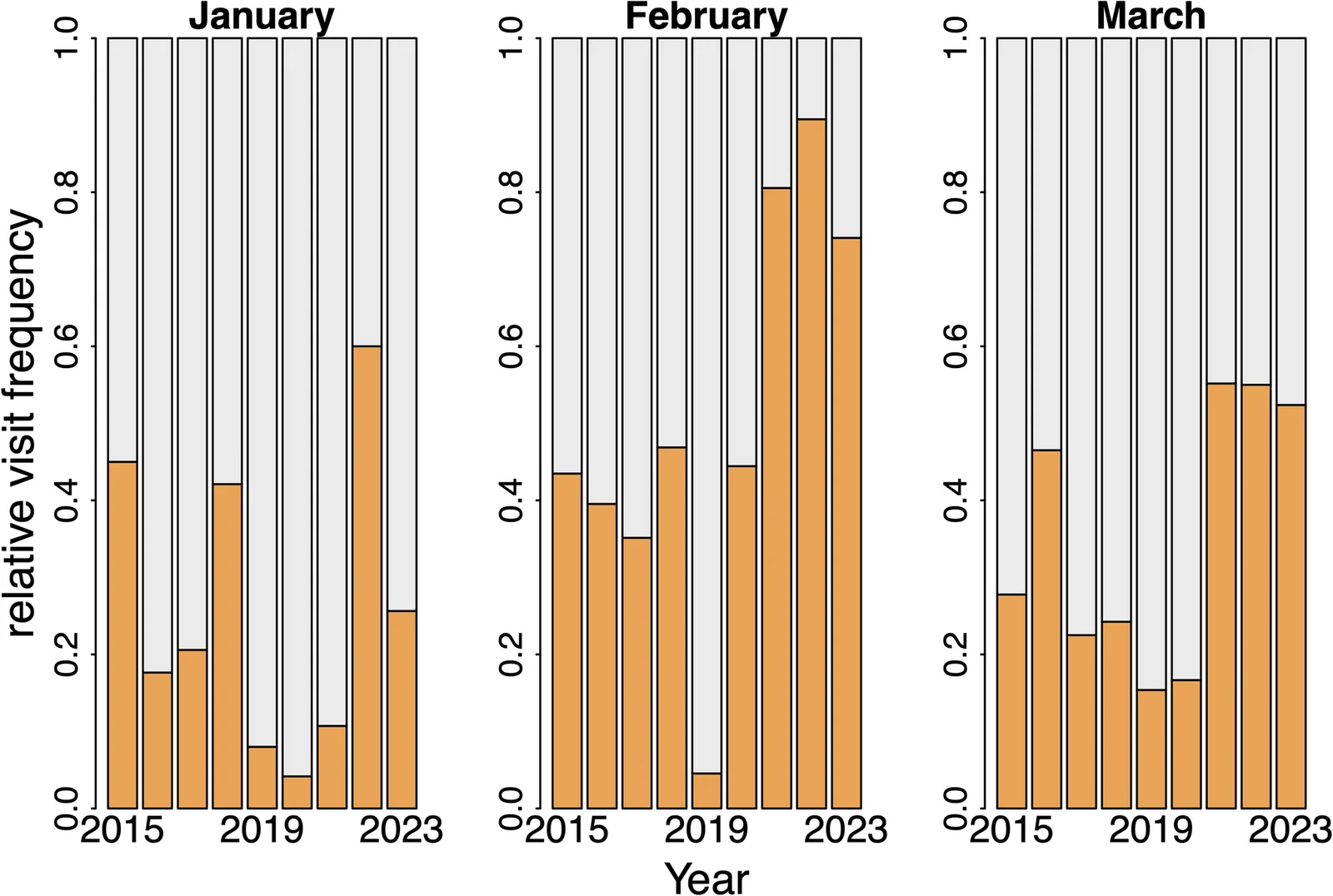
Relative visit frequencies of baboon parties to the target area in January, February and March from 2015 to 2023. Bars represent all days on which a study party was followed in a given month and year. Orange sections indicate the proportion of these days on which at least one waypoint fell within the target area

We compared visit frequencies to the target area from January to March across the study period from 2015 to 2023 to assess changes in space use patterns following the 2019 bushfire. During this period, we collected a total of 815 tracks of seven study parties (Table S2), and Guinea baboon parties visited the target area on 297 days. Visit frequencies in January ranged from 1 to 10, with the number of observation days ranging from 5 to 51 across all years. In February, when ripe *S. spinosa* fruits are typically most abundant, baboons visited the area once in 22 observation days in 2019 compared with 8 to 29 times in the other study years, during which the number of observation days ranged from 18 to 43. In March 2019, the baboons visited the area four times during 26 observation days, with visit frequencies ranging from 4 to 22 and the number observation days ranging from 24 to 43 in the other study years (Fig. 4).

### Linearity and speed of travel (Models 1 and 2)

From April 2014 to March 2023, we collected 2922 tracks, comprising 29,910 location points, for seven study parties. For 530 tracks, at least one waypoint fell into the target area (Table S3). We compared tracks recorded during the fruiting season of *S. spinosa* (January to March) with those recorded during the non-season (April to December). February and March 2019 were classified as non-season due to the loss of ripe fruits following a bushfire. As such, 239 (45.1%) tracks were collected during the season, whereas 291 (54.9%) were collected outside the season. Daily tracks consisted of at least three waypoints, with an average of 11 waypoints (median, range 3 to 15). The analysed approach tracks, including all waypoints from the start of the follow until the first waypoint within the target area, consisted of an average of 6 waypoints (median, range 3 to 14). The average time of arrival in the target area was 09:55 ± 01:11 h (mean ± SD). Among tracks that started at known sleeping sites (n = 313), the mean straight-line distance between sleeping sites and the target area was 2195 m (range 381 to 4295 m). The mean distance travelled from sleeping sites to the target area was 2542 m (range 410 to 7471 m). Guinea baboon parties were subsequently recorded within the target area for an average of 4 waypoints, corresponding to an estimated duration of stay of 01:38 h. However, in many cases (n = 324), observations ended while parties were still within the target area, and thus these values represent minimum stay durations within the target area.

**Fig. 5:**
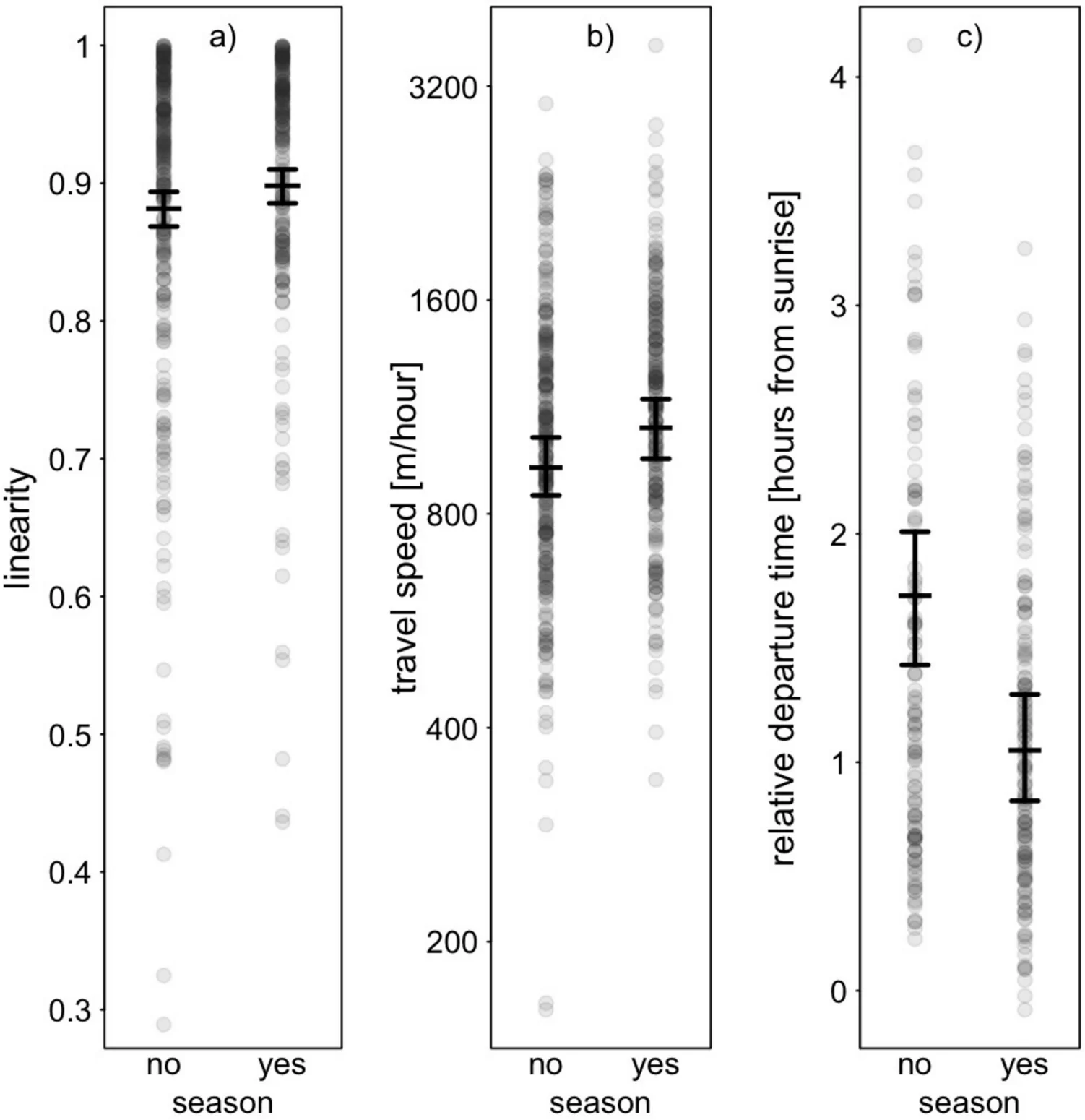
**a**) Linearity, **b**) travel speed, and **c**) relative time of departure from sleeping sites towards the target area in seasons (January to March) vs. non-seasons (April to December). Black lines depict fitted means and 95% confidence intervals. The analysed data span April 2014 to March 2023. February and March 2019 were classified as non-season due to fruit loss following a bushfire

The average linearity of travel paths was 0.89 ± 0.12 (mean, SD, range 0.29–1.00), where values of 1 signify completely linear travel trajectories and values of 0 indicate “meandering” travel. Linearity indices were above 0.8 for 85%, and above 0.9 for 61% of all considered travel paths towards the target area. The linearity of travel towards the target area was higher during the Natal oranges’ season compared to the non-season (Table 2, model 1; Fig. 5a). The Guinea baboon parties travelled at an average speed of 1133 m/h ± 479 (mean, SD), ranging from 160 to 3653 m/h. The baboons travelled faster towards the target area when Natal oranges were in season than when they were not. More specifically, they travelled 14% faster towards the target area when Natal oranges were in season compared to when they were not (Table 2, model 2; Fig. 5b).

**Table 2:** Model results on differences in linearity (Model 1), speed (Model 2), and time of departure (Model 3) between season (January to March) and non-season (April to December) from 2014 to 2023; estimates, standard errors, confidence intervals, significance test, and range of estimates derived from dropping each party one at a time

Model	Term	Estimate	SE	CI _Lower_	CI _Upper_	Test statistic	df	P	Min	Max
1	Intercept	2.006	0.082	1.887	2.129				1.929	2.030
	season.yes^a^	0.170	0.085	0.015	0.313	3.836^c^	1	0.050	0.122	0.257
2	Intercept	6.831	0.051	6.745	6.932				6.798	6.874
	season.yes^a^	0.130	0.037	0.052	0.203	3.528^d^	527.006	<0.001	0.101	0.150
3	Intercept	1.736	0.153	1.426	2.009				1.625	1.811
	season.yes^a^	-0.680	0.138	-0.944	-0.404	-4.913^d^	7.921	0.001	-0.751	-0.601
	z.min.temp^b^	-0.260	0.069	-0.408	-0.126	-3.750^d^	12.327	0.002	-0.294	-0.206

a) Variable dummy coded with the reference level being non-season.

b) z-transformed to a mean of 0 and a standard deviation of 1; mean and SD of the original variable were 21.4 °C and 4.6 °C.

c) χ^2^ statistic from a likelihood-ratio test comparing the full model with the model excluding the test predictor using the function ‘drop1’.

d) t value from t-test for Gaussian models with identity link function.

Data on both departure time from sleeping sites and minimum temperature were available for 285 tracks. Of these, 159 (55.8%) were collected during the season and 126 (44.2%) outside the season. Table 3 summarises the average minimum temperature and time of sunrise during and outside the season.

**Table 3:** Average minimum temperature, time of sunrise, track length, and travel speed towards the target area across time periods (season vs. non-season) from 2014 to 2023

Period	Season	Average minimum temperature (range; °C)	Average time of sunrise (range)	Average track length (25th-75th percentile; m)	Average travel speed (25th-75th percentile; m/h)
Jan to Mar	yes	18.6 (11 to 26.8)	07:12 (06:53 to 07:21)	2476 (1979 to 2810)	1192 (862 to 1443)
Apr to Dec	no	25.0 (15.7 to 31.8)	06:37 (06:27 to 06:58)	2613 (1979 to 3083)	1084 (748 to 1313)

In the mornings, the baboons left their sleeping sites at 08:12 h ± 50 min on average (mean, SD). On average, parties left their sleeping sites 1 h 15 min ± 49 min (mean, SD) after sunrise. The Guinea baboons left their sleeping sites ca. 41 min earlier when travelling towards the target area when fruits of *S. spinosa* were in season vs. when they were not (Table 2; Fig. 5c). The Guinea baboons left their sleeping sites significantly later when minimum temperatures were lower (Table 2, model 3).

## Discussion

Our analyses revealed a substantial shift in space use patterns in the 2019 season compared to other years. This shift was particularly prominent for the February visit frequency, when ripe fruits of *S. spinosa* are usually most abundant. Guinea baboons visited the target area only once in February 2019, whereas the average visit frequency in the years before and after was 16. The lack of visits in February 2019 likely reflects a shift in feeding behaviour following the loss of *S. spinosa* fruits. Although we did not systematically record feeding behaviour in 2019, personal observations suggest that the baboons shifted their feeding behaviour towards *Borassus akeassii* and *Piliostigma reticulatum*, which bear fruit during this period, but occur in other areas of their home ranges (Figure S1). We cannot rule out unobserved visits to the target area (e.g., on days without observations or during afternoons). However, this is unlikely given that typically, the maximum distance from the sleeping sites is reached around noon, and the animals return to their sleeping areas in the afternoon (Ohrndorf et al. 2025a). The visit frequency to the target area in March 2019 was comparable to other years, which may be explained by the presence of other feeding trees in the area that bear ripe fruit in March (e.g., *Bombax costatum*, *Borassus akeassii*, *Piliostigma thonningii*, *Ficus ingens*). These trees are much taller than *S. spinosa* and their fruits are therefore less likely to be impacted by bushfires as heavily as those of *S. spinosa.*

While alternative explanations such as increased predator exposure or reduced shade availability after extensive fires could contribute to reduced visit frequencies, these factors are unlikely to account for the observed pattern either. Bushfires are a regular feature of the habitat, occurring annually (Nielsen et al. 2003), and 2019 differed primarily in the timing and rapid progression of burning rather than in its overall extent. The coincidence of the 2019 bushfires with the ripening period of *S. spinosa* likely caused widespread destruction of ripe fruits while leaving the trees themselves largely intact. NDVI values during the subsequent rainy season were comparable to those of preceding and subsequent years, suggesting no pronounced differences in overall vegetation productivity. Further, studies in other primate species, as well as our own observations, suggest that recently burned areas are often not avoided, but sought out to exploit flushed insects or new vegetation growth (South African vervet monkeys *Chlorocebus pygerythrus*, Herzog et al. 2016; chimpanzees, Pruetz & Herzog 2017; patas monkeys *Erythrocebus patas*, Hanzawa et al. 2024). Taken together, these observations suggest that the destruction of nearly all ripe *S. spinosa* fruits offers the most likely explanation for the reduced visit frequency to the target area in 2019.

The pronounced change in space use patterns indicates that Guinea baboons track the spatio-temporal availability of a high-value food resource within their home range and flexibly adjust space use patterns and foraging decisions in response to changes in resource availability. Such flexibility may be particularly important for animals that live in environments with substantial seasonal variation in food availability (van Schaik et al. 2003; van Schaik 2013). The observed reduction in visit frequency after the loss of ripe *S. spinosa* fruits, followed by the resumption of regular space use patterns in subsequent years, suggests that space use is not purely habitual (de Wit & Dickinson 2009; Dickinson 2012). Instead, baboons appear to take into account current ecological information when making foraging decisions.

One possible explanation for the observed patterns is that certain environmental cues, including perhaps photoperiod, temperature, and the availability of specific food items (Bronson 2009; Janmaat et al. 2012), may contribute to expectations about resource availability in the target area. Once the animals encountered and exploited the resources in the target area, they likely continued to visit the area until the resources were depleted. In 2019, the unexpected absence of ripe fruits, despite previously reliable environmental cues, may have prompted a rapid updating of expectations about food availability within the target area, resulting in the observed changes in space use patterns. This interpretation is consistent with flexible, outcome-sensitive decision-making rather than purely habitual space use (Dickinson 2012). This conclusion is further supported by experimental studies in captivity, in which Guinea baboons readily learned sequential and probabilistic regularities and adjusted their responses based on information acquired in preceding trials or preceding elements of a sequence (Minier et al. 2016; Saldana et al. 2022). Our results complement previous evidence for flexible foraging decisions in other baboon species. While studies have shown that baboons can anticipate high-value food resources and adjust their movements accordingly (e.g., Noser & Byrne 2007b), our study provides additional insight into how such established space use patterns can be adjusted in response to rapid changes in resource availability. Our results suggest that baboons do not rely solely on fixed travel routines but continuously update their foraging decisions based on current ecological information.

In addition, Guinea baboons in the Niokolo-Koba National Park in Senegal travelled more linearly and faster and started earlier toward the target area when *S. spinosa* was in season than when it was not. Travel speed toward the target area increased by about 14%, indicating high motivation and anticipation as study parties travelled toward this high-value food resource. This notion is further supported by earlier departure times from sleeping sites on days when moving toward the target area. The relative time of departure from the sleeping sites was also influenced by the minimum temperature, with later departures occurring at lower minimum temperatures, likely due to thermoregulatory activities (sunbathing) on cold mornings. Interestingly, average minimum temperatures were lower during the season than during the non-season. If earlier departures were solely driven by warmer temperatures, we would expect the opposite pattern than what we observed.

In our analyses, we exclusively assessed movement towards the target area. Within the target area, *S. spinosa* trees occur at very high densities and are relatively evenly distributed, such that from each feeding tree, neighbouring trees are likely easily visible. The target area thus represents an out-of-sight location which baboon parties likely need to navigate towards. Within the target area, however, the abundance and visibility of fruiting trees suggest that space use is better characterised by small-scale foraging decisions rather than by navigational capacities. Given the large approach distances from sleeping sites to the target area, it is unlikely that Guinea baboons relied on olfactory or visual cues from the fruits when navigating towards the target area.

Similar patterns have been observed in other primate species. Chacma baboons approached valuable resources within their home ranges in a fast and linear fashion (Noser & Byrne 2007b). They left sleeping sites earlier when visiting fig trees, a highly preferred food resource, and only returned to less preferred seeds that are harder to ingest in the afternoons. Noser & Byrne (2007b) suggested that this behaviour may be a strategy to optimise the cost and benefits of foraging on a distant depletable, high-value food resource. Similarly, chimpanzee females left their nest sites earlier when breakfasting on preferred ephemeral fruit located far from their nesting sites (Janmaat et al. 2014), and skywalker gibbons (*Hoolock tianxing*) left their sleeping trees earlier when breakfasting on fruit instead of leaves (Fei et al. 2023), most likely to compensate for longer travel times to distant feeding sites as well as to mitigate interspecific competition.

From the available data, we cannot formally infer any specific navigational strategies. Euclidean maps assume highly detailed knowledge of locations and the ability to navigate between them by computing (novel) distances and angles, similar to a coordinate system or a coordinate-based map (Gallistel & Cramer 1996; Di Fiore & Suarez 2007; Janmaat et al. 2021). Topological or route-based maps, in contrast, allow efficient navigation through a set of interconnected, learned travel paths among relevant locations, often facilitated by visual landmarks in the environment (Trapanese et al. 2019; Janmaat et al. 2021). From long-term observations of our study parties, we know they consistently use “bottlenecks” in the landscape (e.g., paths to cross water streams, animal paths, and roads). Some of these “bottlenecks” exist permanently, whereas others form seasonally depending on annual precipitation and water levels. Space use patterns, particularly at these highly conserved landscape features, strongly resemble route-based navigation, as in other baboon species (Sigg 1986; Noser & Byrne 2007a; de Raad & Hill 2019).

In summary, our study provides strong evidence that Guinea baboons track the spatio-temporal availability of a high-value food resource within their home range and flexibly adjust their space use in response to unexpected changes. Rather than following fixed routines, they appear to rapidly update prior experience with current environmental information, suggesting a capacity for adaptive, goal-directed decision-making in a dynamic seasonal environment.

## Supplementary Information

Below is the link to the electronic supplementary material.

Supplementary file1 (PDF 860 kb)

## Data Availability

All data and scripts for analyses are available on Göttingen Research Online (GRO). (https://doi.org/10.25625/Z94QMZ).
