# Supplementary material for: Rapid updating of ecological information in wild Guinea baboons
Source: Anim Cogn. 2026 Oct 30;29(1):79. doi: 10.1007/s10071-026-02119-4 (PMC13722756; doi:10.1007/s10071-026-02119-4)
Supplement: Supplementary file 1 [file 10071_2026_2119_MOESM1_ESM.pdf]

Supplementary material

Rapid updating of ecological information in wild Guinea baboons

Lisa Ohrndorf[1,2], Roger Mundry[1,2,3], Dietmar Zinner[1,2,3], Julia Fischer[1,2,3]

[1] Georg-August-Universität Göttingen, Johann-Friedrich-Blumenbach Institute, Department for Primate Cognition, Kellnerweg 4, 37077 Göttingen, Germany

[2] German Primate Center - Leibniz Institute for Primate Research, Cognitive Ethology Laboratory, Kellnerweg 4, 37077 Göttingen, Germany

3 Leibniz ScienceCampus Primate Cognition, Kellnerweg 4, 37077 Göttingen, Germany

Corresponding author: Lisa Ohrndorf (lohrndorf@dpz.eu)

ORCIDS

Lisa Ohrndorf: 0009-0008-4475-6105

Roger Mundry: 0009-0008-9988-5131

Dietmar Zinner: 0000-0003-3967-8014

Julia Fischer: 0000-0002-5807-0074

Table S1: Number and proportion of 2-minute feeding scans in which an individual was recorded feeding on either of the 11 most frequently consumed plant species between January and March 2022 and 2023, based on behavioural observations.

Plant species	January		February		March		Total	
	Count	%	Count	%	Count	%	Count	%
*Borassus akeassii**	269	33	62	9.2	67	12.3	398	19.5
Piliostigma thonningii	2	0.2	46	6.8	236	43.3	284	13.9
Wissadula rostrata	180	22.1	78	11.5	21	3.9	279	13.7
*Strychnos spinosa**	40	4.9	123	18.2	85	15.6	248	12.2
Piliostigma reticulatum	147	18	26	3.8	2	0.4	175	8.6
Hibiscus asper	21	2.6	64	9.5	3	0.6	88	4.3
Grass	33	4	40	5.9	11	2	84	4.1
Acacia seyal	0	0	27	4	46	8.4	73	3.6
*Celtis integrifolia**	4	0.5	26	3.8	9	1.7	39	1.9
Terminalia macroptera	12	1.5	16	2.4	7	1.3	35	1.7
Cochlospermum tinctorium	0	0	24	3.6	0	0	24	1.2

* Plant species that produce fleshy fruit consumed by Guinea baboons in Simenti. Note that these fruits differ substantially in size and nutritional composition. For more information see the Supplementary Material in Zinner et al. (2021).

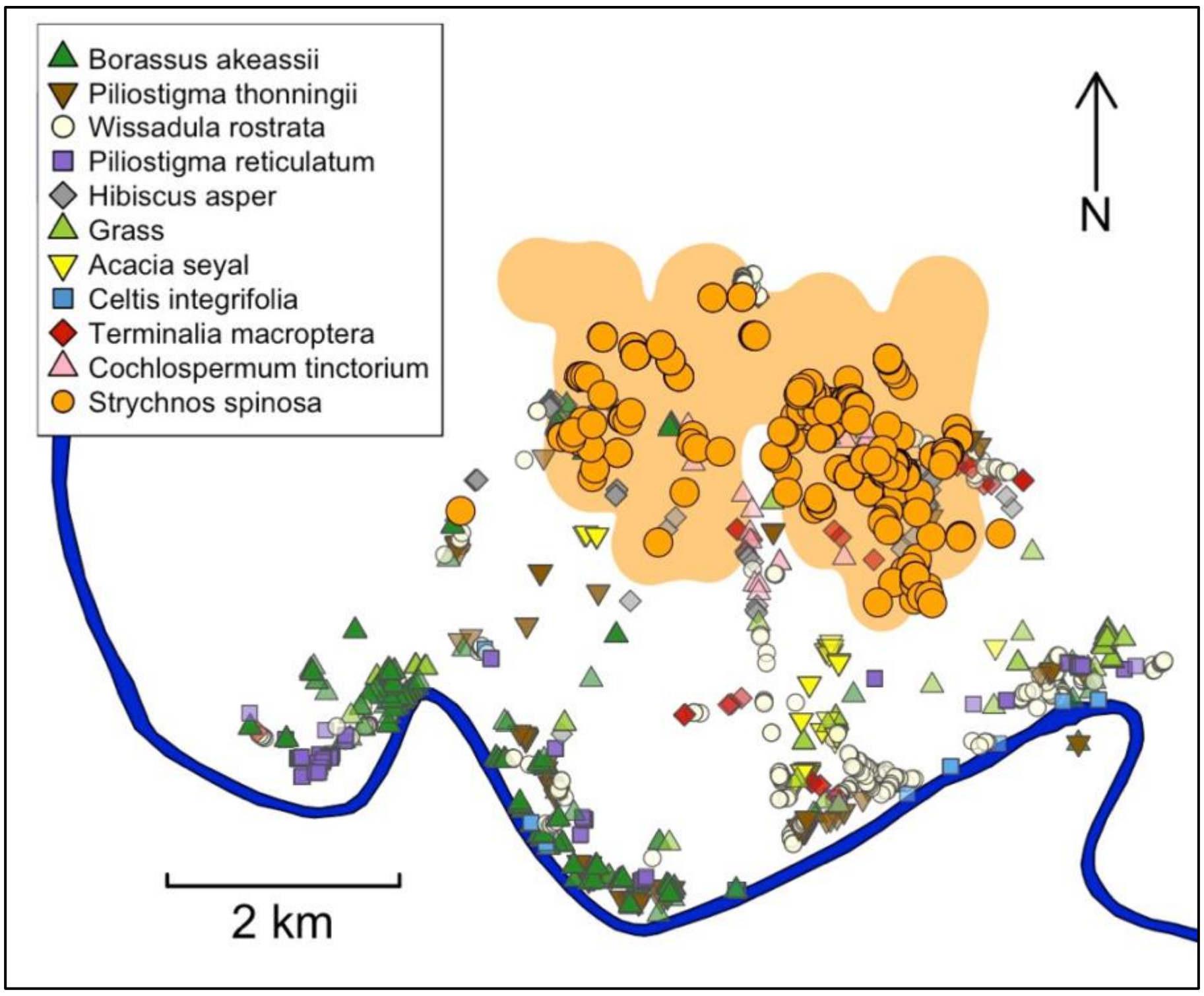

Figure S1: Locations of feeding records for the 11 most frequently consumed plant species from January to March of 2022 and 2023, based on behavioural observations. The Gambia River is depicted in blue. The orange polygon marks the target area in which *Strychnos spinosa* is particularly abundant.

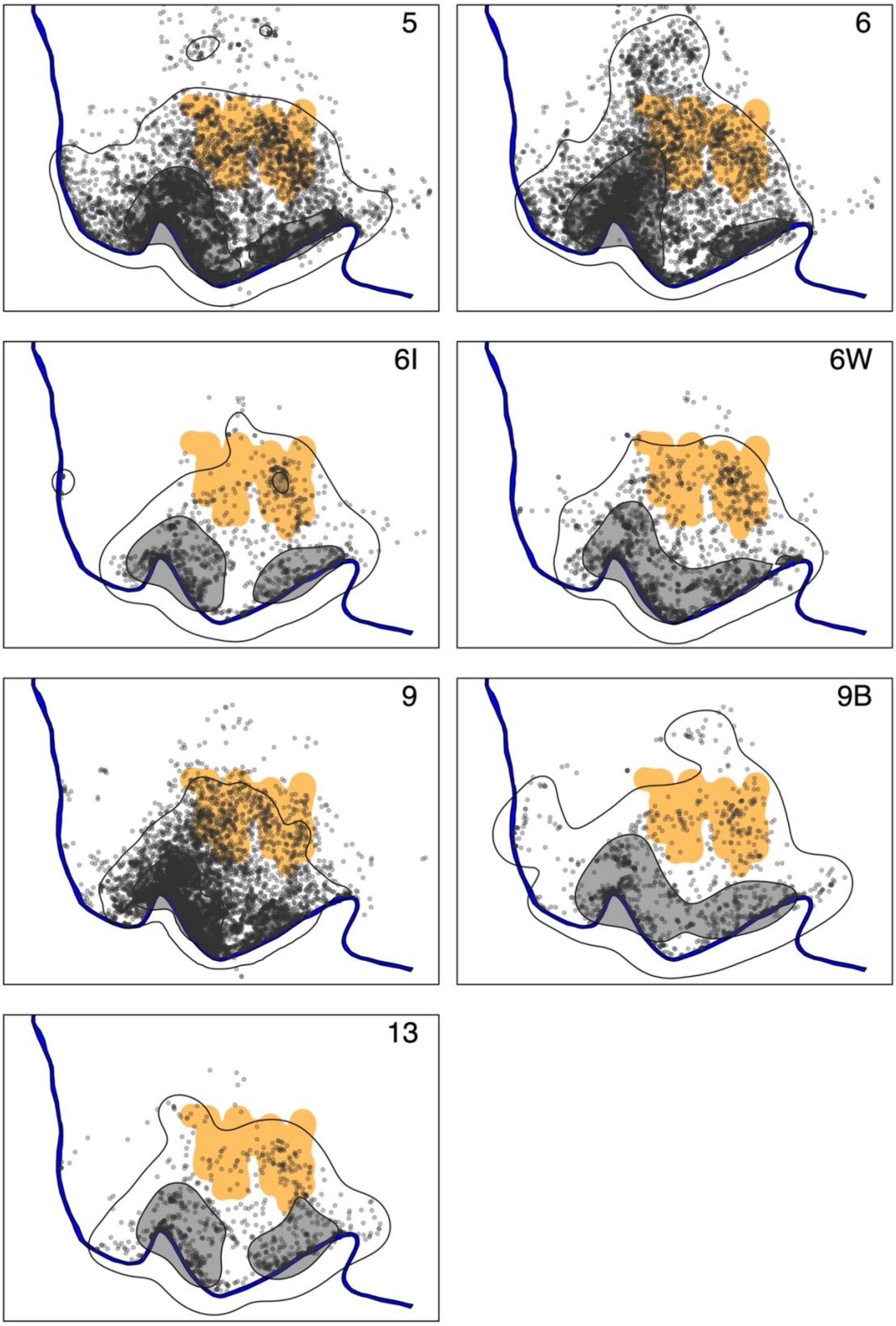

Figure S2: Home ranges (95% contour level, transparent polygon) and core areas (50% contour level, grey polygon) of the 7 study parties from 2015 to 2023. The target area is shown as an orange polygon, and the Gambia River is depicted as a blue line.

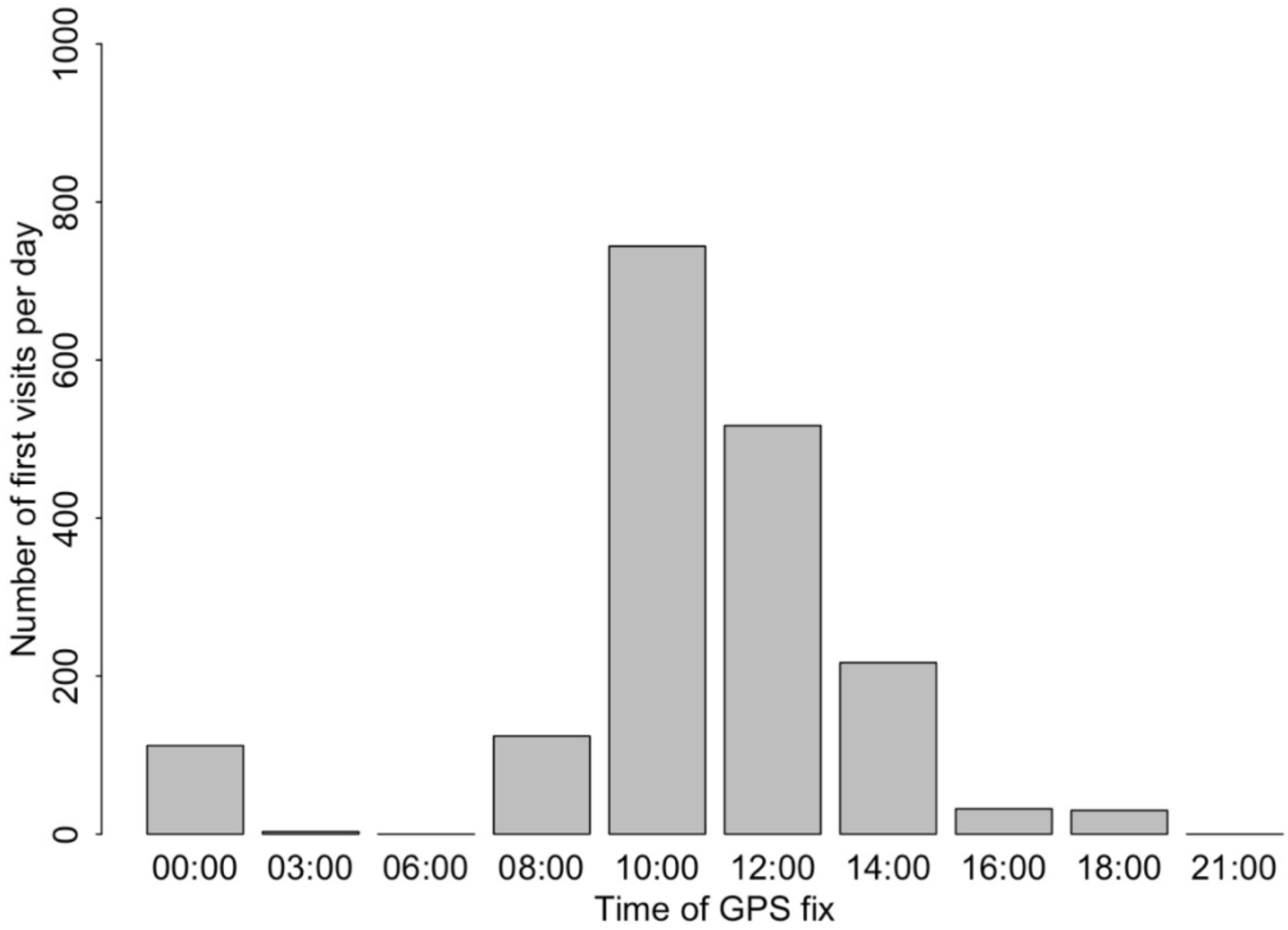

Figure S3: Number of first GPS fixes per animal and day that fell into the target area by time of day (n = 1779). The presented data were collected by equipping adult Guinea baboons with GPS collars from 2010-2012 and in 2022. The collars were programmed to take locational fixes every two hours during the day (06:00 to 18:00) and an additional three fixes during the night to mark the location of the sleeping site (21:00, 00:00, 03:00). For detailed information on the capture and collaring procedure see Knauf et al. (2015) and Ohrndorf et al. (2025).

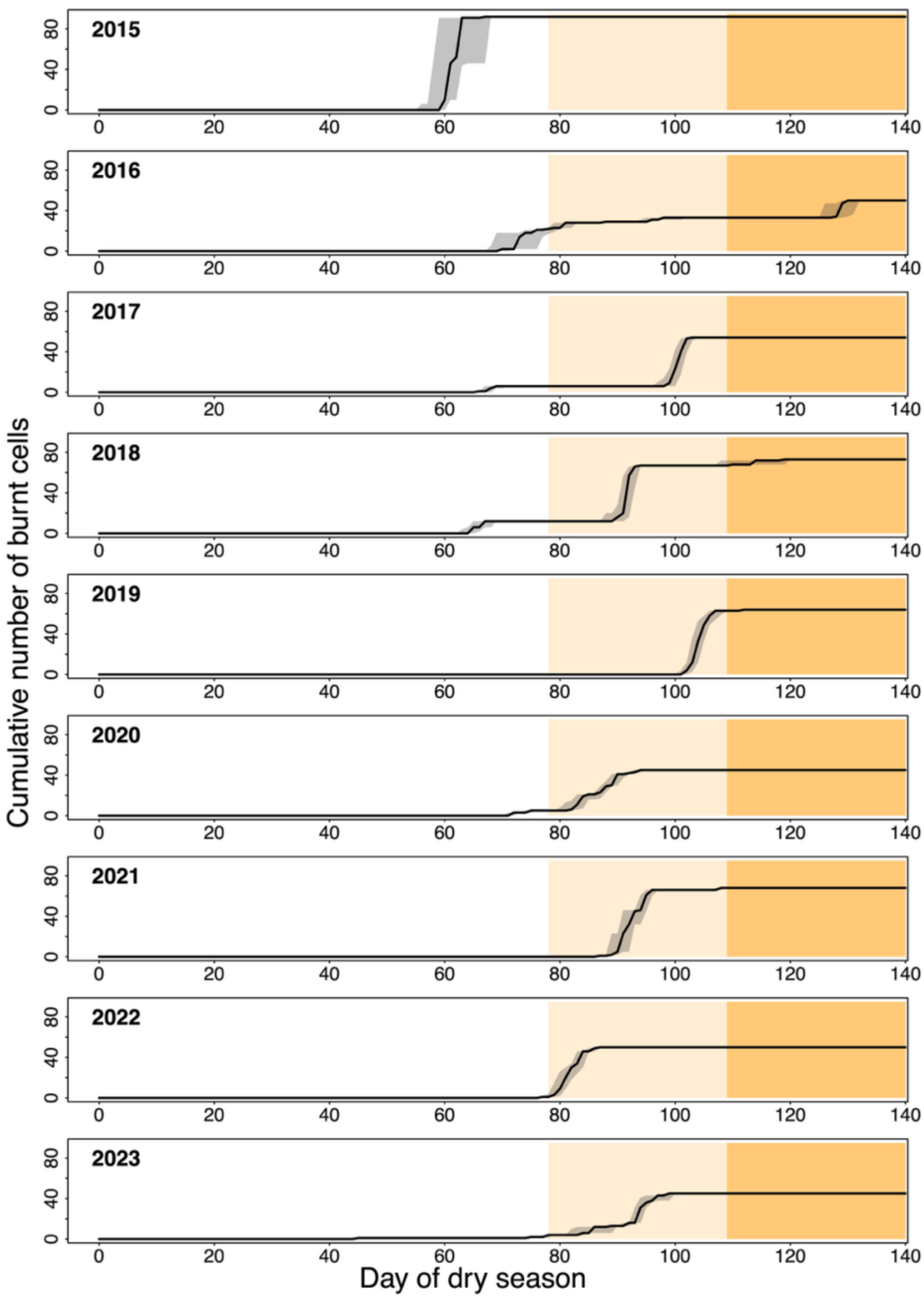

Figure S4: Timing and extent of fires within the target area and a surrounding 500 m buffer during the dry seasons from 2015 to 2023. The black lines indicate the cumulative number of burnt cells in the target area and the 500 m buffer. The year refers to the dry season (15 October of the previous year to 30 May of the indicated year). Day of dry season illustrates the progression of the season starting on 15 October. The light orange area denotes January, when fruits of *S. spinosa* begin to ripen, and the dark orange area denotes February, when ripe fruits are most abundant. Grey shaded areas indicate the uncertainty in the estimated fire date as extracted from the MODIS burned-area product MCD64A1 Version 6.1 (Giglio et al. 2021).

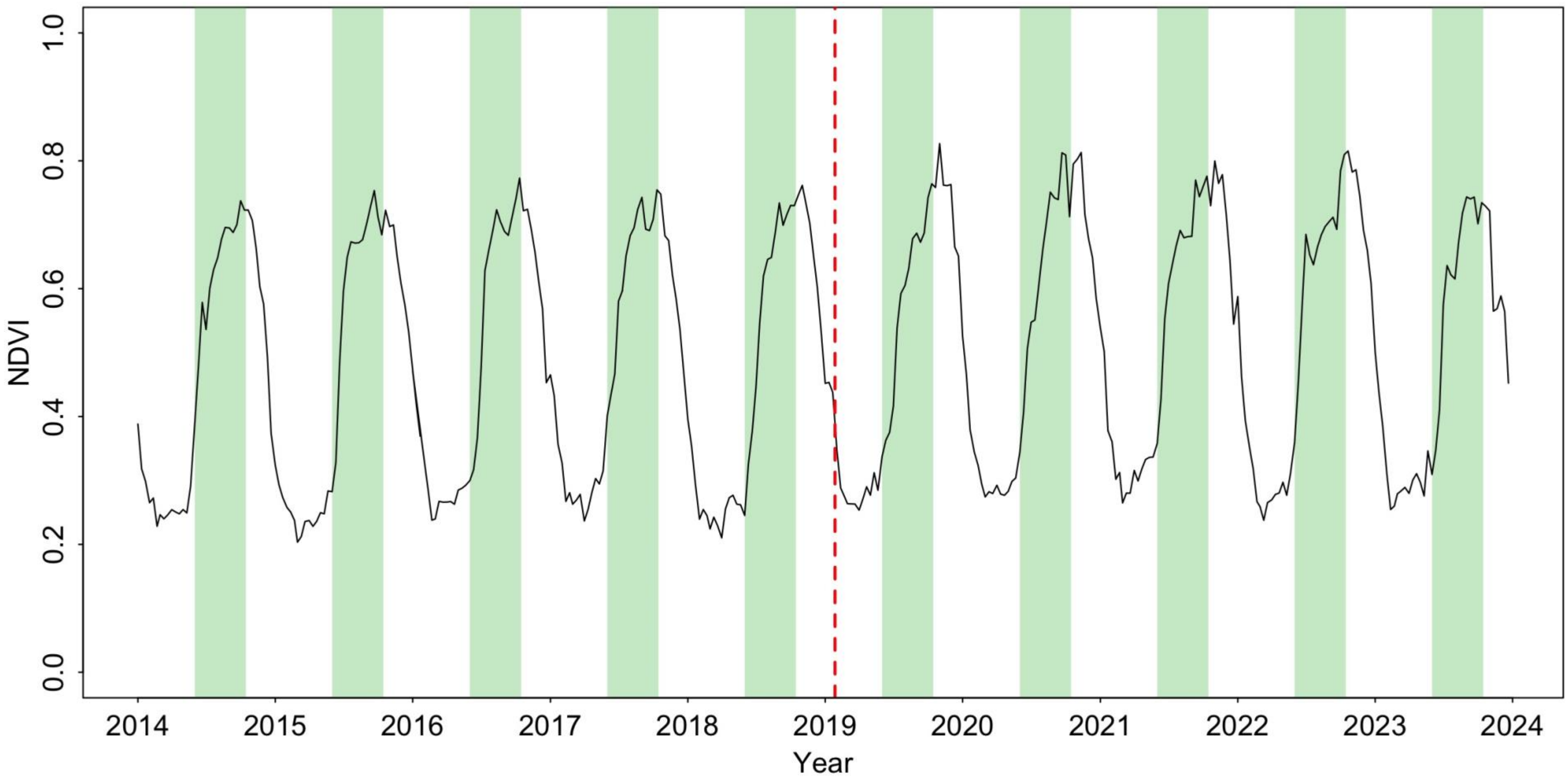

Figure S5: Average NDVI values of the target and a surrounding 500 m buffer (13.0263–13.0662° N, 13.2907–13.2346° W) from 2014 to 2023 extracted from version 3 of the Copernicus Global Land Service (Copernicus Global Land Service, 2020). Green shaded areas depict the rainy season (June to mid-October). The red dashed line marks 27 January 2019, when the majority of cells overlapping with the target area burned.

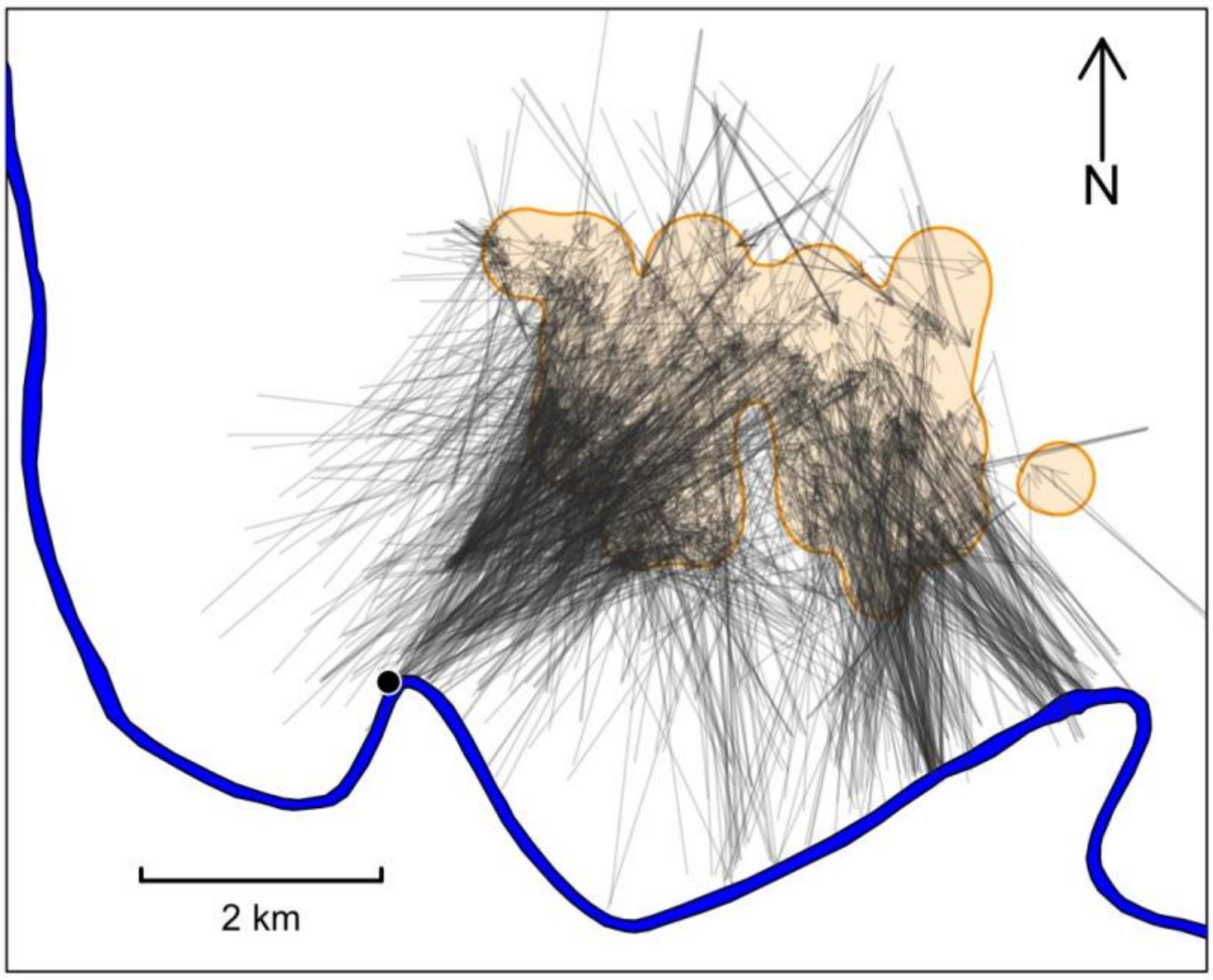

Figure S6: Approach directions to the first waypoint within the target area, depicted as vectors connecting the previous GPS fix and the first GPS fix within the target area for each individual and day (*n* = 1779). In only 66 of 1779 cases (3.7%), the previous GPS location was located north of the target area, indicating that approaches from the north were uncommon. The presented data were collected from adult Guinea baboons equipped with GPS collars between 2010 and 2012 and in 2022. Collars were programmed to record locations every two hours during the day (06:00–18:00) and an additional three fixes during the night to mark the location of the sleeping site (21:00, 00:00, 03:00). For detailed information on the capture and collaring procedure, see Knauf et al. 2015, Ohrndorf et al. 2025.

Table S2: Number of daily party tracks collected from January to March for each year, with (Y) or without (N) visits to the target area.

Year	N	Y	Total
2015	50	29	79
2016	91	46	137
2017	82	29	111
2018	53	31	84
2019	66	7	73
2020	53	13	66
2021	45	48	93
2022	22	42	64
2023	56	52	108

Table S3: Number of tracks towards the target area per study party during and outside of the season of *S. spinosa*. In total, 530 tracks that consisted of at least 3 waypoints before entering the target area were available across parties and years.

Party	season	
	no	yes
5	81	80
6	77	60
6I	10	15
6W	16	26
9	87	41
9B	13	8
13	7	9

Table S4: Total number of GPS tracks collected per study party across the study period (2014 to 2023), including the number and proportion of tracks containing at least one waypoint within the target area.

Party	Total tracks	Total visits	Visit proportion
5	772	229	0.297
6	714	220	0.308
6I	112	39	0.348
6W	154	51	0.331
9	763	177	0.232
9B	110	33	0.300
13	88	29	0.330

Table S5: Estimated standard deviations and correlations of random effects for all fitted models. Note that models 1 and 2 did not include estimates of the correlation between random intercept and slope.

Model	Grouping factor	Term 1	Term 2	SD / correlations
1	Party	Intercept		0.041
	Party	season.yes		<0.001
2	Party	Intercept		0.097
	Party	season.yes		<0.001
3	Party	Intercept		0.333
	Party	season.yes		0.153
	Party	z.min.temp		0.081
	Party	Intercept	season.yes	-0.671
	Party	Intercept	z.min.temp	-0.785
	Party	season.yes	z.min. emp	0.067

References

Copernicus Global Land Service. 2020. *Normalized Difference Vegetation Index (NDVI) 300 m, global, 10-daily – Version 3*. European Commission, Copernicus Global Land Service. https://doi.org/10.2909/905223f4-2c3d-4cb6-ad8c-d6d065707465

Giglio L, Justice C, Boschetti L, Roy D (2021). *MODIS/Terra+Aqua Burned Area Monthly L3 Global 500m SIN Grid V061 [Data set]*. NASA Land Processes Distributed Active Archive Center. https://doi.org/10.5067/MODIS/MCD64A1.061

Knauf S, Barnett U, Maciej P, Klapproth M, Ndao I, Frischmann S, Fischer J, Zinner D, Liu H (2015). High prevalence of antibodies against the bacterium Treponema pallidum in Senegalese Guinea baboons (*Papio papio*). *PLoS One*, 10(11), e0143100. https://doi.org/10.1371/journal.pone.0143100

Ohrndorf L, Mundry R, Beckmann J, Fischer J, Zinner D (2025). Impact of food availability and predator presence on patterns of landscape partitioning among neighbouring Guinea baboon (*Papio papio*) parties. *Movement Ecology*, 13(1), 9. https://doi.org/10.1186/s40462-025-00534-9

Zinner D, Klapproth M, Schell A, Ohrndorf L, Chala D, Ganzhorn JU, Fischer J (2021). Comparative ecology of Guinea baboons (*Papio papio*). *Primate biology*, 8(1), 19-35. https://doi.org/10.5194/pb-8-19-2021
